# Extracellular Vesicles From Hyperammonemic Rats Induce Neuroinflammation in Cerebellum of Normal Rats: Role of Increased TNFα Content

**DOI:** 10.3389/fimmu.2022.921947

**Published:** 2022-07-13

**Authors:** Paula Izquierdo-Altarejos, Mar Martínez-García, Vicente Felipo

**Affiliations:** Laboratory of Neurobiology, Príncipe Felipe Research Centre, Valencia, Spain

**Keywords:** neuroinflammation, hyperammonemia, extracellular vesicles, glial activation, Purkinje neurons, GABAergic neurotransmission

## Abstract

Hyperammonemia plays a main role in the neurological impairment in cirrhotic patients with hepatic encephalopathy. Rats with chronic hyperammonemia reproduce the motor incoordination of patients with minimal hepatic encephalopathy, which is due to enhanced GABAergic neurotransmission in cerebellum as a consequence of neuroinflammation. Extracellular vesicles (EVs) could play a key role in the transmission of peripheral alterations to the brain to induce neuroinflammation and neurological impairment in hyperammonemia and hepatic encephalopathy. EVs from plasma of hyperammonemic rats (HA-EVs) injected to normal rats induce neuroinflammation and motor incoordination, but the underlying mechanisms remain unclear. The aim of this work was to advance in the understanding of these mechanisms. To do this we used an ex vivo system. Cerebellar slices from normal rats were treated ex vivo with HA-EVs. The aims were: 1) assess if HA-EVs induce microglia and astrocytes activation and neuroinflammation in cerebellar slices of normal rats, 2) assess if this is associated with activation of the TNFR1-NF-kB-glutaminase-GAT3 pathway, 3) assess if the TNFR1-CCL2-BDNF-TrkB pathway is activated by HA-EVs and 4) assess if the increased TNFα levels in HA-EVs are responsible for the above effects and if they are prevented by blocking the action of TNFα. Our results show that ex vivo treatment of cerebellar slices from control rats with extracellular vesicles from hyperammonemic rats induce glial activation, neuroinflammation and enhance GABAergic neurotransmission, reproducing the effects induced by hyperammonemia *in vivo*. Moreover, we identify in detail key underlying mechanisms. HA-EVs induce the activation of both the TNFR1-CCL2-BDNF-TrkB-KCC2 pathway and the TNFR1-NF-kB-glutaminase-GAT3 pathway. Activation of these pathways enhances GABAergic neurotransmission in cerebellum, which is responsible for the induction of motor incoordination by HA-EVs. The data also show that the increased levels of TNFα in HA-EVs are responsible for the above effects and that the activation of both pathways is prevented by blocking the action of TNFα. This opens new therapeutic options to improve motor incoordination in hyperammonemia and also in cirrhotic patients with hepatic encephalopathy and likely in other pathologies in which altered cargo of extracellular vesicles contribute to the propagation of the pathology.

## Introduction

Sustained peripheral inflammation is associated to neuroinflammation and cognitive and motor impairment in different pathologies such as rheumatoid arthritis, diabetes, hyperammonemia, hepatic encephalopathy and in neurodegenerative diseases such as multiple sclerosis, Parkinson’s or Alzheimer’s diseases (1–5)

There are several mechanisms by which peripheral alterations in the immune system may be transmitted to the brain to induce neuroinflammation, including infiltration of peripheral immune cells into the brain (6, 7), activation by peripheral interleukins such as IL-6, IL-1β or IL-17 of their receptors in endothelial cells and transmission of the signal into the brain (8), or activation by peripheral cytokines of vagal nerve which transmits the peripheral alterations to the brain (9). There is increasing evidence that another main mechanism by which peripheral alterations are transmitted to the brain is by infiltration of peripheral extracellular vesicles with altered cargo, which transmit pathological signals to the brain (10, 11). This has been reported for example for Parkinson’s disease, Amyotrophic lateral sclerosis or autism (12–15).

Patients with liver cirrhosis show sustained hyperammonemia and peripheral inflammation which act synergistically to induce minimal hepatic encephalopathy, with mild cognitive and motor impairment and motor incoordination (2, 3, 16). These neurological alterations are reproduced in rats with chronic hyperammonemia, that show peripheral inflammation, which induces neuroinflammation in cerebellum and hippocampus leading to altered GABAergic and glutamatergic neurotransmission which induce motor incoordination and cognitive impairment (3).

Motor incoordination is due to enhanced GABAergic neurotransmission in cerebellum (17, 18), which in turn is due to neuroinflammation (19–21). Neuroinflammation increases TNFα in cerebellum, which enhances activation of its receptor TNFR1, leading to translocation to the nucleus of NF-kB, which increases glutaminase levels, leading to increased extracellular glutamate. This glutamate is taken up, together with Na^+^, by glutamate transporters in activated astrocytes. This alters the Na^+^ gradient and reverses the function of the GABA transporter GAT3, whose amount and membrane expression are increased in hyperammonemia. This leads to increased extracellular GABA and GABAergic neurotransmission which is responsible for motor incoordination in hyperammonemia and hepatic encephalopathy (20).

Increased activation of TNFR1 also increases the levels of CCL2 in cerebellum, which is responsible for activation of microglia, which in turn increases BDNF levels. Increased BDNF levels enhance activation of TrkB in Purkinje neurons leading to increased membrane expression of KCC2, which enhances the transmembrane chloride gradient, further enhancing GABAergic neurotransmission (22).

The induction of neuroinflammation and neurological impairment in hyperammonemic rats is mediated by peripheral alterations, as indicated by the fact that they are prevented by preventing peripheral inflammation (2). We have recently shown that extracellular vesicles (EVs) play a key role in the transmission of these peripheral alterations to the brain (23). Extracellular vesicles from plasma of hyperammonemic rats (HA-EVs) show altered cargo, with increased levels of TNFα and of its receptor TNFR1. When injected to normal rats, these HA-EVs reach the cerebellum, especially Purkinje neurons and microglia, and induce neuroinflammation and motor incoordination (23). However, the mechanisms by which HA-EVs induce activation of microglia and astrocytes and motor incoordination remain unclear. The aim of this work was to advance in the understanding of these mechanisms. To do this we used an ex vivo system. Cerebellar slices from normal rats were treated ex vivo with HA-EVs. The aims of these studies were to assess if HA-EVs induce activation of microglia and astrocytes and neuroinflammation in cerebellar slices of normal rats ex vivo, to evaluate if this is associated with activation of the TNFR1-NF-kB-glutaminase-GAT3 pathway, and to analyze if the TNFR1-CCL2-BDNF-TrkB pathway is also increased by HA-EVs. We also aimed to investigate whether the increased levels of TNFα in HA-EVs are responsible for the above effects and if they are prevented by blocking the action of TNFα.

## Materials and Methods

### Model of Chronic Hyperammonemia

Male Wistar rats (6–7 weeks old, Charles River Laboratories) were made hyperammonemic by feeding them with an ammonium containing diet during 4-5 weeks as described by Felipo et al. (24). All the experiments were approved by the Comite de Experimentación y Bienestar Animal (CEBA) of our Center and by Conselleria de Agricultura of Generalitat Valenciana and were performed in accordance with guidelines of the Directive of the European Commission (2010/63/EU) for care and management of experimental animals.

### Isolation of Extracellular Vesicles

Extracellular vesicles were obtained by ultracentrifugation from plasma of hyperammonemic rats (after 4-5 weeks of diet). Plasma from hyperammonemic rats treated with Infliximab (an anti-TNFα antibody) in a previous study of the group (2) were also used to obtain extracellular vesicles. Plasma (3 mL) was thawed on ice, diluted to 20 mL with sterile PBS and centrifuged at 2000 g for 20 min at 4°C. Supernatants were collected, filtered through 0.22 um sterile filters and centrifuged at 150,000 g for 16 hours at 4°C. Supernatants were removed and pellets were washed by ultracentrifugation at 150,000 g for 2 hours and 110,000 g for 70 min at 4°C. Pellets were resuspended in 100 μL of PBS, protein quantity was determined by bicinchoninic acid method and samples were stored at −80°C until use.

### Study Design

Hyperammonemic rats after 4-5 weeks of hyperammonemic diet and control rats of the same age were sacrificed by decapitation to extract the brain (n=18 per group). Cerebelli were dissected and immediately immersed into ice-cold Krebs buffer (NaCl 119 mM, NaHCO_3_ 26.2 mM, glucose 11 mM, KCl 2.5 nM, CaCl_2_ 2.5 mM, KH_2_PO_4_ 1 mM aerated with 95% O_2_ and 5% CO_2_ at pH 7.4). Cerebellar slices of 400 μm were obtained with a vibratome and incubated in the wells of a perfusion system (Campden Instruments) for 15 min at 35.5°C in Krebs buffer for stabilization. Once stabilized, the slices from control rats were incubated during 30 min at 35.5°C with the following treatments: 10 µg/mL of Infliximab (Remicade) (C+Inflix), 10 ng/mL of recombinant TNFα (Abcam) (C+rec TNFα), 10 µg/mL of extracellular vesicles from hyperammonemic rats (C+HA-EVs), 10 µg/mL of EVs from hyperammonemic rats previously incubated with 10 µg/mL of Infliximab (Remicade) for 1 hour at 37°C (C+HA-EVs+Inflix); 10 µg/mL of extracellular vesicles from hyperammonemic rats treated with Infliximab *in vivo* (C+HAInflix-EVs). Cerebellar slices from control and hyperammonemic rats incubated in Krebs buffer without treatment were included as reference (C and HA, respectively).

### Analysis of Protein Content by Western Blot

After the ex vivo treatments, slices were homogenized by sonication for 20 s in buffer (Tris-HCl 66 mM pH 7.4, SDS 1%, EGTA 1 mM, glycerol 10%, leupeptin 0.2 mg/mL, NaF 1 mM, Na orto-vanadate 1 mM). Homogenates were subjected to electrophoresis and immunoblotting as described by Felipo et al. (24). Primary antibodies used were against BDNF (1:1000, Invitrogen), CCL2 (1:1000, Proteintech), Glutaminase 1 (1:1000, Novus Biologicals), GAT3 (1:1000, Synaptic Systems), HMGB1 (1:1000, Abcam), KCC2 (1:1000, Millipore), TNFα (1:500, RD systems), TNFR1 (1:500, Abcam) and TrkB (1:500, Abcam). Anti-actin (1:5000, Abcam) was used as protein loading control. Secondary antibodies were anti-rabbit, anti-rat, or anti-goat IgG conjugated with alkaline phosphatase (1:4000, Sigma). Membranes were scanned and band intensities were quantified using Alpha Imager 2200 version 3.1.3.

### Analysis of Membrane Expression by Cross-Linking With BS3

After the incubation ex vivo, slices were added to tubes containing ice-cold Krebs buffer with or without 2 mM BS3 (Rockford) and incubated for 30 min at 4°C. Cross-linking was terminated by quenching the reaction with 100 mM glycine during 10 min at 4°C. The slices were homogenized as above and analyzed by immunoblotting using the primary antibodies: CCR2 (1:1000, Novus), GAT3 (1:1000, Synaptic Systems), KCC2 (1:1000, Millipore), TNFR1 (1:500, Abcam) and TrkB (1:500, Abcam). Membrane expression was calculated as the difference between the intensity of the bands without BS3 (total protein) and with BS3 (non-membrane protein).

### Immunohistochemistry

For immunohistochemistry staining, cerebellar slices were fixed in 4% paraformaldehyde in 0.1 M phosphate buffer (pH 7.4) during 24 h at 4°C after ex vivo incubation with the different treatments. Immunohistochemistry was performed as in Arenas et al. (25). Samples were embedded in paraffin and 5 μm sections were cut and mounted onto coated glass slides. Sections were hydrated, incubated with 3% H_2_O_2_ for 15 min to block endogenous peroxidases, and with 6% normal goat serum for 1 h. Sections were then incubated with primary antibodies against Iba1 (1:300, Wako), GFAP (1:400; Sigma), TNFα (1:300, Abcam) and Glutaminase 1 (1:100, Novus Biologicals) overnight at 4°C. Next, sections were incubated with secondary biotinylated antibodies goat anti-rabbit and goat anti-mouse (1:200, Vector Laboratories) for 1 h at room temperature. Then, sections were incubated with ABC complex (Vector Laboratories) during 30 min, followed by diaminobenzidine (DAB substrate kit, Abcam) until desired color was acquired (for a maximum of 10 min). Finally, sections were counterstained with hematoxylin (Dako). Sections were scanned with an Aperio Versa system (Leica Biosystems).

### Analysis of Microglia and Astrocytes Activation

From the immunohistochemistry scans, images at 40x magnification were taken using the software ImageScope64 (8-10 fields per rat and area of study). Microglia activation was analyzed both in molecular layer and white matter, while astrocytes activation was analyzed in white matter. Activated microglia acquired an ameboid shape, reducing its processes and its area. The area of individual Iba1 stained cells was calculated using IpWin 32 software and the average of all cells analyzed for each rat was assessed, considering a reduction of the average area as a measure of activation. For astrocytes activation, the percentage of each image stained by GFAP was determined using ImageJ software and the results were expressed as percentage of control. In this case, higher percentage of area covered by GFAP was considered as higher activation of the astrocytes.

### Quantification of TNFα and Glutaminase Content in Purkinje Neurons

From the immunohistochemistry scans, images at 40x magnification including Purkinje neurons were taken using the software ImageScope64 (8 fields per rat). Neurons were manually outlined using the ROI manager function in ImageJ and four squared regions per image were taken and considered as background. Inverted values of mean gray value were recorded, and the mean intensity of each neuron was calculated as: mean intensity of the neuron – average intensity of the background regions.

### Analysis of NF-κB Activation in Purkinje Neurons by Immunofluorescence

NF-κB activation was analyzed in the paraffin-embedded sections from the ex vivo experiment as in Dadsetan et al. (26). For immunofluorescence, primary antibodies were NF-κB p50 (1:200, Abcam) and Iba1 (1:300, Abcam), followed by donkey anti-mouse Alexa 488 and donkey anti-rabbit Alexa 647 secondary antibodies (1:400, Invitrogen) and DAPI staining. Slides were observed under a confocal microscope (Leica TCS-SP2-AOBS) at 63x magnification and 8 fields per rat were acquired.

Nuclear and cytoplasmic intensity of p50 subunit was analyzed using ImageJ. Nuclei were outlined using the ROI manager function on DAPI blue channel and the selection was applied on the green channel (p50) to measure fluorescence. Mean intensity for each nucleus was measured. For cytoplasmic analysis of p50 subunit of NF-κB, green channels were used, and cytosol of each cell was manually outlined using a freehand selection of ImageJ and mean intensity recorded. Results are expressed as nuclear/cytoplasmic ratio of p50 subunit of NF-κB. Number of microglial cells (red signal) expressing NF-κB (green signal) was manually counted.

### Statistical Analysis

Data are expressed as mean ± SEM. All statistical analyses were performed using GraphPad Prism software 8.1.2 version. Data were analyzed by one-way analysis of variance (ANOVA) followed by Tukey *post hoc* test. A confidence level of 95% was accepted as significant. Data of immunohistochemistry and immunofluorescence analysis are the average of all cells (Iba1, TNFα and Glutaminase staining) or all fields (GFAP staining) analyzed per each rat.

## Results

We have previously shown that HA-EVs reach the cerebellum after intravenous injection, induce neuroinflammation *in vivo*, with microglia and astrocytes activation, and impair motor coordination (23). However, the mechanisms by which peripheral vesicles transmit pathological effects to the brain remain unclear. To study these mechanisms, we performed ex vivo studies in which we incubated cerebellar slices from control rats with HA-EVs, showing that this also induces neuroinflammation and glial activation (see below). We analyzed the underlying mechanisms and the role played by TNFα in the HA-EVs. We assessed if these effects are prevented by blocking TNFα in these vesicles or by adding vesicles from plasma of hyperammonemic rats treated *in vivo* with Infliximab (an anti-TNFα). We also assessed if addition of recombinant TNFα reproduced the effects of HA-EVs.

### Extracellular Vesicles From Hyperammonemic Rats Induce a TNFα-Mediated Microglia and Astrocytes Activation in Cerebellar Slices From Control Rats

**Figure 1:**
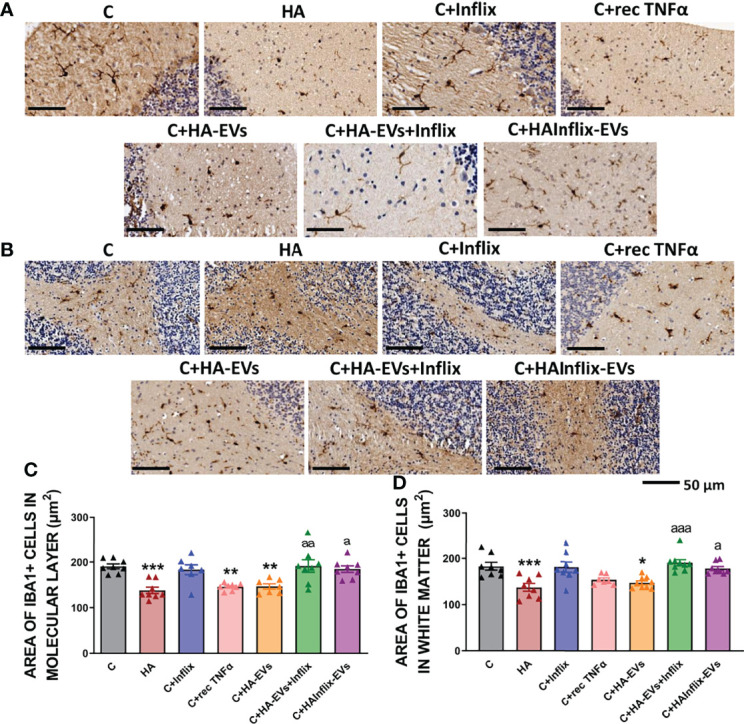
Microglia activation after ex vivo incubation of cerebellar slices from control rats with extracellular vesicles from hyperammonemic rats. Representative images of the immunohistochemistry against Iba1 in cerebellar slices after the ex vivo incubation in **(A)** molecular layer and **(B)** white matter. Area of the microglia (Iba1 positive cells) in **(C)** molecular layer (n=7-8) and **(D)** white matter (n=7-9). Sample size indicates number of animals. One-way ANOVA with Tukey *post-hoc* test was performed to compare all groups. Values are the mean ± SEM. Values significantly different from controls are indicated by asterisk (*p<0.05, **p<0.01, ***p<0.001) and values significantly different from C+HA-EVs group are indicated by a (a=p<0.05, aa=p<0.01, aaa=p<0.001). Scale bar = 50 µm. Experimental groups: cerebellar slices from control (C) and hyperammonemic rats (HA) incubated in basal conditions. Cerebellar slices from control slices incubated with the following treatments: the anti-TNFα Infliximab (C+Inflix); recombinant TNFα (C+rec TNFα); extracellular vesicles from hyperammonemic rats (C+HA-EVs); extracellular vesicles from hyperammonemic rats previously incubated with Infliximab (C+HA-EVs+Inflix); extracellular vesicles from hyperammonemic rats treated *in vivo* with Infliximab (C+HAInflix-EVs).

The area of microglial cells was reduced (p<0.001) in the molecular layer (**Figures 1A, C**) and white matter (**Figures 1B, D**) of cerebellar slices from hyperammonemic rats, indicating microglial activation. Ex vivo incubation of cerebellar slices from control rats with HA-EVs produces a similar activation of microglia (**Figures 1A–D**). Blocking TNFα in the HA-EVs with anti-TNFα (infliximab) prevents their effects. Activation of microglia by HA-EVs was prevented if the HA-EVs were pre-incubated with anti-TNFα (Infliximab; p < 0.05). Moreover, EVs isolated from HA rats treated *in vivo* with Infliximab, which do not show increased TNFα content, do not induce activation of microglia (**Figures 1A–D**), addition of recombinant TNFα to cerebellar slices from control rats also induced microglial activation. These data show that HA-EVs induce microglial activation in the ex vivo system in cerebellar slices from control rats and that this effect is mediated by the enhanced levels of TNFα in the HA-EVs.

**Figure 2:**
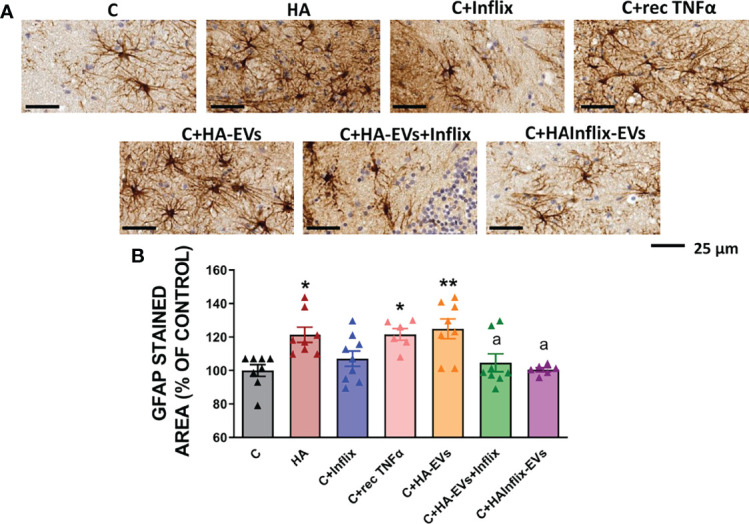
Astrocytes activation after ex vivo incubation of cerebellar slices from control rats with extracellular vesicles from hyperammonemic rats. **(A)** Representative images of the immunohistochemistry against GFAP in cerebellar slices (white matter) after the ex vivo incubation. **(B)** Area stained with GFAP expressed as percentage of controls (n=6-9). Sample size indicates number of animals. One-way ANOVA with Tukey *post-hoc* test was performed to compare all groups. Values are the mean ± SEM. Values significantly different from controls are indicated by asterisk (*p<0.05, **p<0.01) and values significantly different from C+HA-EVs group are indicated by a (a = p<0.05). Scale bar = 25 µm.

Similar effects were observed on astrocytes activation (**Figure 2**). Cerebellar slices from hyperammonemic rats show increased astrocytes activation, as reflected in the increased area stained with GFAP (p < 0.05). A similar activation of astrocytes was induced by addition to cerebellar slices from control rats of HA-EVs ex vivo (**Figures 2A, B**). As for microglia activation, astrocytes activation did not occur if HA-EVs were pre-incubated with Infliximab, or if they were isolated from HA rats treated with Infliximab, indicating a key role of TNFα in this process. Incubation of control slices with recombinant TNFα also induced astrocytes activation (p < 0.05) (**Figures 2A, B**).

### Extracellular Vesicles From Hyperammonemic Rats Induce a TNFα-Mediated Activation of the TNFR1-CCL2-BDNF-TrkB-KCC2 Pathway in Cerebellar Slices From Control Rats

We have recently shown that microglia activation in cerebellum of hyperammonemic rats is mediated by enhanced membrane expression and activation of TNFR1, which increases CCL2 production in Purkinje neurons which in turn increases activation and CCR2 membrane expression, leading to increased BDNF in microglia. BDNF released from activated microglia increases activation and membrane expression of TrkB which, in turn, enhances membrane expression of the chloride co-transporter KCC2, increasing the chloride gradient and GABAergic neurotransmission (22, see **Figure 7**).

**Figure 3:**
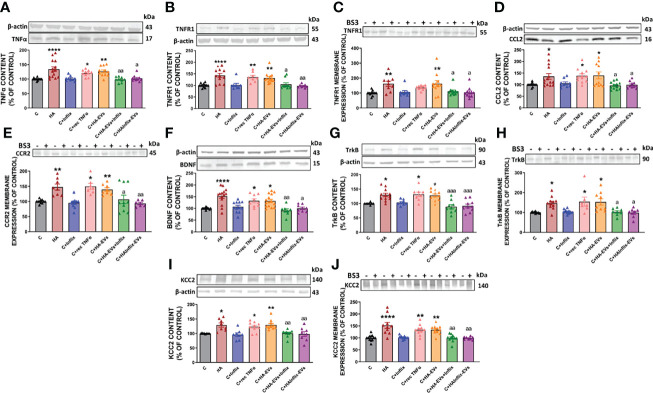
Extracellular vesicles from hyperammonemic rats activate CCL2-CCR2-TrkB-KCC2 pathway in cerebellar slices from control rats. Protein content of **(A)** TNFα (n=9-14), **(B)** TNFR1 (n=8-13), **(D)** CCL2 (n=9-14), **(F)** BDNF (n=9-15), **(G)** TrkB (n=8-12) and **(I)** KCC2 (n=8-10) measured by Western blot in cerebellar slices. Membrane expression of **(C)** TNFR1 (n=9-13), **(E)** CCR2 (n=7-11), **(H)** TrkB (n=7-11) and **(J)** KCC2 (n=8-12) measured by Western blot in cerebellar slices incubated with or without BS3. Sample size indicates number of animals. Representative images of Western blot bands and loading control are shown and molecular weight of each protein is indicated. Values are expressed as percentage of control and are the mean ± SEM. Values significantly different from controls are indicated by asterisk (*p<0.05, **p<0.01, ****p<0.001) and values significantly different from C+HA-EVs group are indicated by a (a = p<0.05, aa = p<0.01, aaa = p<0.001).

We assessed if addition of HA-EVs ex vivo to cerebellar slices of control rats also induces activation of this TNFR1-CCL2-BDNF-TrkB-KCC2 pathway and if this effect is mediated by the enhanced levels of TNFα in the HA-EVs. As shown in **Figure 3**, HA-EVs increased in cerebellar slices from control rats the content of TNFα (**Figure 3A**); of its receptor TNFR1 (**Figure 3B**); the membrane expression of TNFR1 (**Figure 3C**); the content of CCL2 (**Figure 3D**); membrane expression of the CCL2 receptor CCR2 (**Figure 3E**); the content of BDNF (**Figure 3F**); the content (**Figure 3G**) and membrane expression (**Figure 3H**) of the BDNF receptor TrkB; and the content (**Figure 3I**) and membrane expression (**Figure 3J**) of KCC2. These data show that addition of HA-EVs ex vivo to cerebellar slices of control rats reproduces the activation of the TNFR1-CCL2-BDNF-TrkB-KCC2 pathway induced by hyperammonemia.

Moreover, activation of the TNFR1-CCL2-BDNF-TrkB-KCC2 pathway did not occur if HA-EVs were pre-incubated with Infliximab, or if they were isolated from HA rats treated with Infliximab, indicating a key role of TNFα in this process. Incubation of control slices with recombinant TNFα also activates this pathway (**Figures 3A–J**).

### Extracellular Vesicles From Hyperammonemic Rats Induce a TNFα-Mediated Activation of the TNFR1-NF-kB-Glutaminase-GAT3 Pathway in Cerebellar Slices From Control Rats

Another pathway which contributes to enhance GABAergic neurotransmission in cerebellum of hyperammonemic rats is the TNFR1-NF-kB-glutaminase-GAT3 pathway (20). Increased membrane expression and activation of TNFR1 induces nuclear translocation and activation of the transcription factor NF-kB, which increases the content of glutaminase and of extracellular glutamate. This in turns leads to increased uptake of glutamate and Na^+^ by glutamate transporters in activated astrocytes, resulting in altered Na^+^ gradient which reverses the function of the GABA transporter GAT3, leading to release of GABA to the extracellular fluid, which enhances GABAergic neurotransmission. Hyperammonemia also increases the content and membrane expression of GAT3, further enhancing extracellular GABA and GABAergic neurotransmission (20).

We assessed if addition of HA-EVs ex vivo to cerebellar slices of control rats also induces activation of this TNFR1-NF-kB-glutaminase-GAT3 pathway and if this effect is mediated by the enhanced levels of TNFα in the HA-EVs. As already mentioned above, HA-EVs increase the content (**Figure 3B**) and membrane expression (**Figure 3C**) of TNFR1 in cerebellar slices from control rats. We assessed if this is associated with increased nuclear translocation and activation of NF-kB.

**Figure 4:**
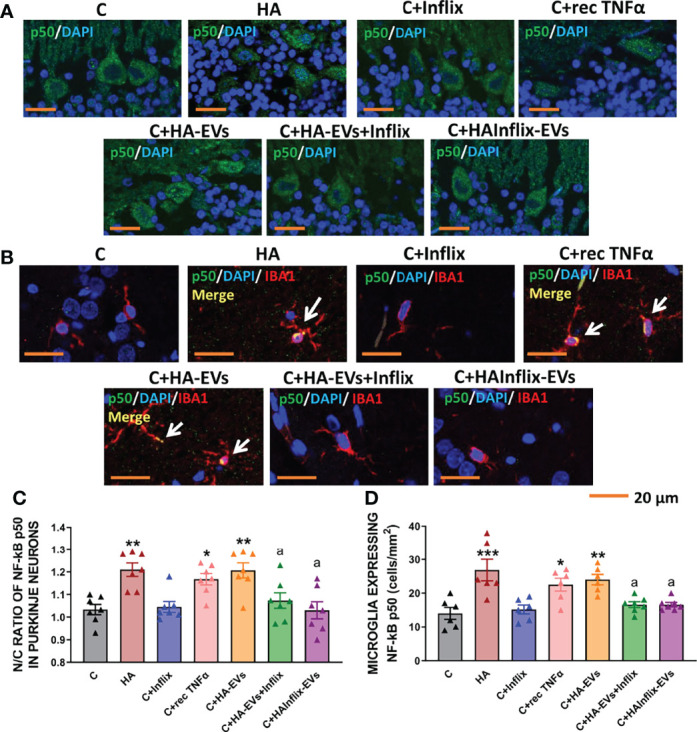
Extracellular vesicles from hyperammonemic rats induce NF-κB activation in Purkinje neurons and microglia in cerebellar slices from control rats. Representative images of **(A)** immunofluorescence against NF-κB p50 subunit (green) and **(B)** double immunofluorescence against NF-κB p50 subunit (green) and Iba1 (red). **(C)** Ratio of Nuclear/Cytoplasmic NF-κB p50 subunit in Purkinje neurons (n=6-7). **(D)** Number of microglial cells expressing NF-κB p50 subunit in white matter of the cerebellum, measured by double immunofluorescence and expressed as cells/mm^2^ (n=7). Sample size indicates number of animals. One-way ANOVA with Tukey *post-hoc* test was performed to compare all groups. Values are the mean ± SEM. Values significantly different from controls are indicated by asterisk (*p<0.05, **p<0.01, ***p<0.001) and values significantly different from C+HA-EVs group are indicated by a (a=p<0.05). Scale bar = 20 μm.

HA-EVs induced a translocation of the p50 subunit of NF-kB to the nucleus of Purkinje neurons, similar to that induced by hyperammonemia *per se* (**Figures 4A, C**). Moreover, HA-EVs also increased the number of microglial cells expressing NF-kB (**Figures 4B, D**), thus reproducing the activation of NF-kB induced by hyperammonemia *in vivo*.

Nuclear translocation and activation of NF-kB did not occur if HA-EVs were pre-incubated with Infliximab, or if they were isolated from HA rats treated with Infliximab, indicating a key role of TNFα in this process. Incubation of control slices with recombinant TNFα also activates NF-kB (**Figures 4A–D**).

**Figure 5:**
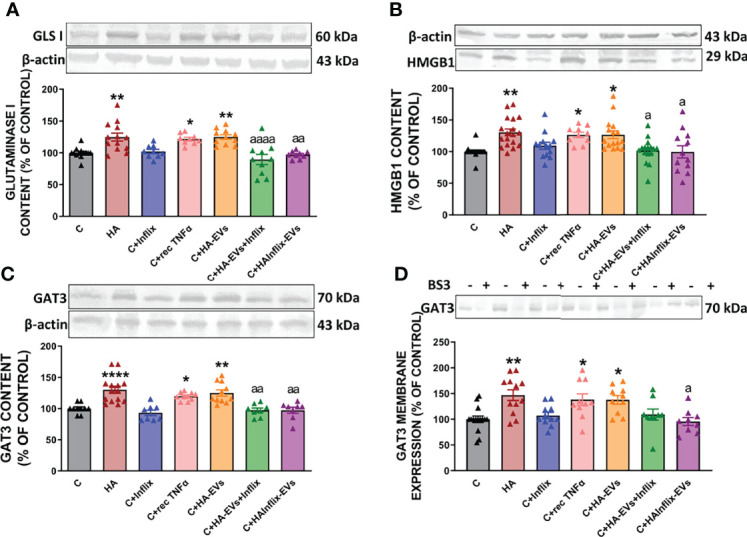
Extracellular vesicles from hyperammonemic rats activate glutaminase-GAT3 pathway in cerebellar slices from control rats. Protein content of **(A)** glutaminase I (n=9-13), **(B)** HMGB1 (n=10-18) and **(C)** GAT3 (n=8-14) measured by Western blot in cerebellar slices. **(D)** Membrane expression (n=9-15) of GAT3 measured by Western blot in cerebellar slices incubated with or without BS3. Representative images of Western blot bands and loading control are shown and molecular weight of each protein is indicated. Sample size indicates number of animals. Values are expressed as percentage of control and are the mean ± SEM. Values significantly different from controls are indicated by asterisk (*p<0.05, **p<0.01, ****p<0.001) and values significantly different from C+HA-EVs group are indicated by a (a = p<0.05, aa = p<0.01, aaaa = p<0.0001).

We then analyzed if increased activation of NF-kB by HA-EVs is associated with increased levels of proteins which transcription is modulated by this factor. HA-EVs induced in cerebellar slices from control rats an increase in the content of TNFα (**Figure 3A**), glutaminase I (**Figure 5A**) and of HMGB1 (**Figure 5B**), which did not occur if HA-EVs were pre-incubated with Infliximab, or if they were isolated from HA rats treated with Infliximab. Incubation of control slices with recombinant TNFα also increased glutaminase I and HMGB1 content (**Figures 5A, B**).

Moreover, the increased content of glutaminase I in cerebellar slices from control rats treated ex vivo with HA-EVs is associated with increased content (**Figure 5C**) and membrane expression (**Figure 5D**) of GAT3, which were also prevented by blocking TNFα with infliximab and were reproduced by recombinant TNFα.

**Figure 6:**
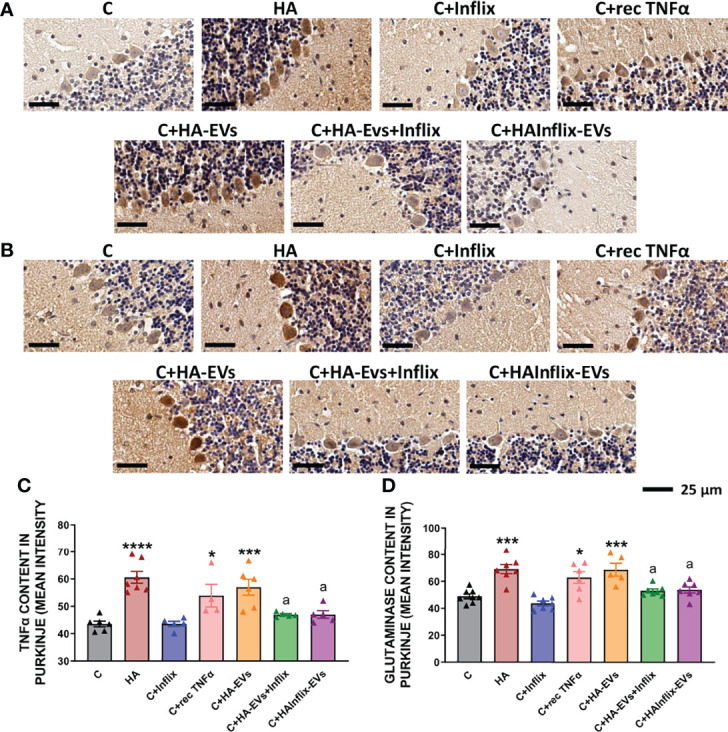
Extracellular vesicles from hyperammonemic rats increase TNFα and glutaminase content in Purkinje neurons in cerebellar slices from control rats. Representative images of immunohistochemistry against **(A)** TNFα and **(B)** glutaminase, showing Purkinje neurons of the cerebellum. Content of **(C)** TNFα (n=4-7) and **(D)** glutaminase (n=5-8) in Purkinje neurons, measured as mean intensity. Sample size indicates number of animals. One-way ANOVA with Tukey *post-hoc* test was performed to compare all groups. Values are the mean ± SEM. Values significantly different from controls are indicated by asterisk (*p<0.05, **p<0.01, ***p<0.001, ****p<0.0001) and values significantly different from C+HA-EVs group are indicated by a (a=p<0.05). Scale bar = 25 μm.

To confirm that activation of the TNFR1-NF-kB pathway increases TNFα and glutaminase I in Purkinje neurons, we analyzed their content in these neurons by immunohistochemistry (**Figure 6**). Both TNFα (**Figures 6A, C**) and glutaminase I (**Figures 6B, D**) content was increased by HA-EVs in Purkinje neurons of control rats. These increases did not occur if HA-EVs were pre-incubated with Infliximab, or if they were isolated from HA rats treated with Infliximab. Incubation of control slices with recombinant TNFα also increased TNFα and glutaminase I content in Purkinje neurons (**Figures 6A–D**).

## Discussion

Sustained peripheral inflammation leads to cognitive and motor impairment in many pathological situations including neurodegenerative diseases such as multiple sclerosis, Parkinson’s and Alzheimer’s diseases as well as pathologies such as diabetes, rheumatoid arthritis, hyperammonemia and hepatic encephalopathy (1–5). However, how peripheral inflammation induces these neurological alterations is not well understood. There are several mechanisms by which peripheral alterations may be transmitted to the brain.

There is increasing evidence that EVs play a key role in the transmission of pathological signals from the periphery to the brain in different pathologies. For example, injection to mice of EVs from serum of patients with Parkinson’s disease induces neuroinflammation and motor impairment in these mice (27). Peripheral EVs also play a key role in the transmission to the brain of pathological signals in hyperammonemia and hepatic encephalopathy. We have recently shown that injection to normal rats of HA-EVs induces neuroinflammation and motor incoordination (23).

**Figure 7:**
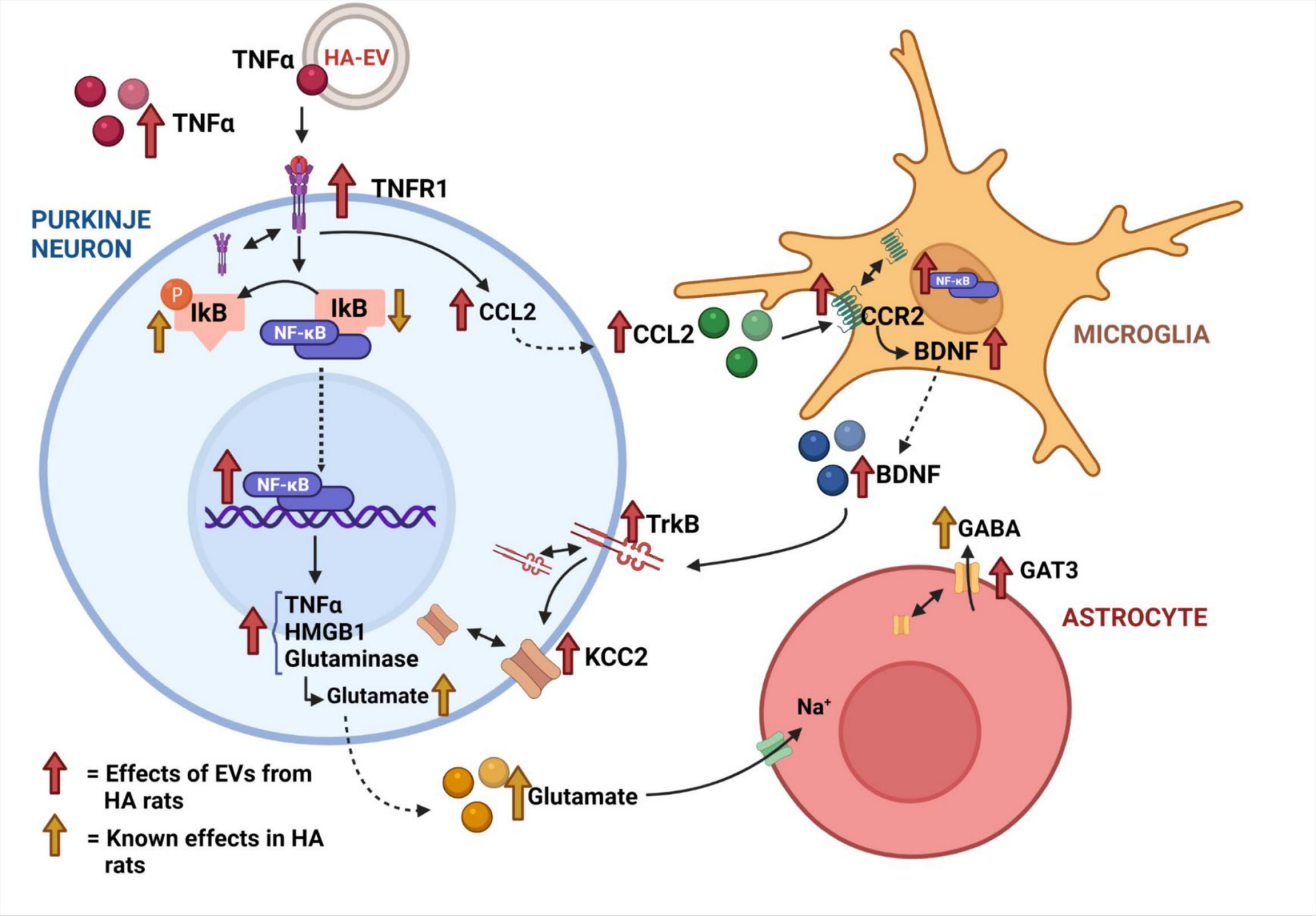
Summary of the ex vivo effects of extracellular vesicles from hyperammonemic rats on cerebellar slices from control rats.

However, how peripheral EVs transmit the pathology to the brain is not well known. The cargo of EVs (proteins, miRNAs, mRNAs) is altered in these pathological situations (23, 27) and it is likely that some of the altered compounds mediate the alterations induced by EVs in brain. We have now studied the mechanisms by which EVs from plasma of hyperammonemic rats (HA-EVs) trigger the alterations in cerebellum which would mediate the induction of motor incoordination reported by Izquierdo-Altarejos et al. (23). We have identified the mechanisms by which HA-EVs induce these alterations and show that the increased content of TNFα in these EVs play a key role in triggering these mechanisms. The mechanisms involved are summarized in **Figure 7**.

HA-EVs increase in cerebellar slices from control rats the content of TNFα and the content and membrane expression of its receptor TNFR1, resulting in increased activation of TNFR1. This induces two main effects which will contribute to enhance GABAergic neurotransmission and induce motor incoordination: the increase in CCL2 and the nuclear translocation of the transcription factor NF-kB. The increase in CCL2 triggers microglia activation in cerebellum of hyperammonemic rats, which is prevented by blocking its receptor CCR2 (22). We show now that HA-EVs also increases CCL2 content in cerebellum of control rats and this is associated with microglia activation (**Figure 7**), thus reproducing the effects found in hyperammonemia. Moreover, these effects are triggered by TNFα in the HA-EVs and are prevented by blocking the action of TNFα with infliximab. This identifies a main mechanism by which HA-EVs induce microglia activation and neuroinflammation in cerebellum.

In hyperammonemia and hepatic encephalopathy peripheral inflammation induces neuroinflammation, which is prevented by preventing the appearance of peripheral inflammation (2, 26 and 28). This neuroinflammation enhances GABAergic neurotransmission in cerebellum by two main mechanisms: increasing the TNFR1-CCL2-BDNF-TrkB-KCC2 pathway (22) and the TNFR1-NF-kB-glutaminase-GAT3 pathway (3 and 20). We show here that HA-EVs also increase these pathways, which would enhance GABAergic neurotransmission and induce motor incoordination as reported by Izquierdo-Altarejos et al. (23).

The increased content of CCL2 induced by HA-EVs in cerebellar slices from control rats is associated with increased membrane expression and activation of its receptor CCR2 which increases BDNF in activated microglia (**Figure 7**), in agreement with the effects reported in cerebellum of hyperammonemic rats by Arenas et al. (22). The increased levels of BDNF result in increased content, membrane expression and activation of its receptor TrkB (**Figure 7**). Increased activation of TrkB, in turn, enhances membrane expression of the chloride co-transporter KCC2 (**Figure 7**) as also reported by Arenas et al. (22) in hyperammonemic rats. KCC2 is expressed in neurons, especially in Purkinje neurons in cerebellum (29, 30) and transport chloride ions from the inside to the outside of the neurons, thus reducing intracellular Cl^-^ concentration (31–33). The increased membrane expression of KCC2 induced by HA-EVs would result in larger transmembrane chloride gradient in Purkinje neurons. Activation of GABA_A_ receptors leads to the opening of their chloride channel, thus allowing the flux through it of chloride ions. The magnitude of the flux and of the responses to activation of GABA_A_ receptors depends on the chloride gradient (31–33). The increase in the membrane expression of KCC2 and in the transmembrane chloride gradient induced by HA-EVs would enhance GABA_A_ receptors response and GABAergic neurotransmission, thus contributing to the induction of motor incoordination.

Increased membrane expression and activation of TNFR1 in cerebellar slices from control rats treated with HA-EVs also induces the nuclear translocation and activation of NF-kB, which in turn increases the content of glutaminase I (**Figure 7**). This agrees with the effects reported by Cabrera-Pastor et al. (20) in cerebellum of hyperammonemic rats. They showed that increased membrane expression and activation of TNFR1 induces nuclear translocation and activation of the transcription factor NF-kB, which increases the content of glutaminase and of extracellular glutamate. This in turns leads to increased uptake of glutamate and Na^+^ by glutamate transporters in activated astrocytes, resulting in altered Na^+^ gradient which reverses the function of the GABA transporter GAT3, leading to release of GABA to the extracellular fluid, which enhances GABAergic neurotransmission. Hyperammonemia also increases the content and membrane expression of GAT3, further enhancing extracellular GABA and GABAergic neurotransmission (20, 21). We show here that HA-EVs also induce in cerebellum of control rats an increase in TNFR1 content and membrane expression, nuclear translocation and activation of NF-kB, increased content of glutaminase, especially in Purkinje neurons, and increased content and membrane expression of GAT3 (**Figure 7**). Activation of this pathway would also contribute to the induction of motor incoordination by HA-EVs reported by Izquierdo-Altarejos et al. (23), by increasing extracellular GABA as shown by Cabrera-Pastor et al. (20). It has been shown that the levels of extracellular GABA are increased in cerebellum of hyperammonemic rats and that correlate with and contribute to motor incoordination in hyperammonemic rats (3, 17, 20, 21 and 34).

Enhanced GABAergic neurotransmission has been also reported in patients with liver cirrhosis and hepatic encephalopathy, which also show sustained hyperammonemia (35). Moreover, the authors suggest that GABAergic neurotransmission is mainly enhanced in Purkinje neurons and correlate with ataxia. The results reported here show that EVs from plasma of hyperammonemic rats are enough to induce neuroinflammation and enhance GABAergic neurotransmission. EVs may also contribute to the enhanced GABAergic neurotransmission in cerebellum and motor incoordination in patients with cirrhosis and hepatic encephalopathy through mechanisms similar to those reported here.

The data reported also show that the increased levels of TNFα in the HA-EVs are the main trigger of the activation of the TNFR1-CCL2-BDNF-TrkB-KCC2 and the TNFR1-NF-kB-glutaminase-GAT3 pathways which result in microglial activation, enhanced GABAergic neurotransmission and motor incoordination. This provides a target for therapeutic intervention by blocking the action of TNFα; for example, with antibodies against TNFα such as infliximab, which is already being used in clinical practice to reduce peripheral inflammation in diseases such as rheumatoid arthritis or Crohn’s disease (36, 37). In fact, we have already shown that treatment of rats with hyperammonemia and hepatic encephalopathy due to portacaval shunts with infliximab prevents the induction of neuroinflammation, of alterations in neurotransmission and of motor and cognitive impairment (2, 26 and 28). The results reported here unveil a main mechanism by which peripheral treatment with infliximab prevents these deleterious effects. The EVs from hyperammonemic rats treated with infliximab do not induce neuroinflammation, microglia activation or GABAergic neurotransmission enhancement when injected to normal rats. This would be due to the lack of increase in the content of TNFα in these EVs, in contrast with the remarkable increase observed in EVs from hyperammonemic rats not treated with infliximab. Treatment with infliximab (or other anti-TNFα reagents) may also prevent the pathogenic changes in the cargo of EVs in other pathologies, thus helping to reduce neuroinflammation and neurological impairment. This could be the mechanism by which infliximab treatment induces the improvement in cognitive function in patients with rheumatoid arthritis or sarcoidosis reported by Raftery et al. (38) and Elfferich et al. (39).

Increased content of TNFα in the membrane of EVs seems to contribute to the propagation of other pathologies. For example, Gao et al. (40) reported that exosomes derived from mature dendritic cells increase endothelial inflammation and atherosclerosis *via* membrane TNFα mediated NF-κB pathway activation. Söderberg et al. (41) showed that exosomes released by melanoma cells contain TNFα which generate higher reactive oxygen species levels in neighboring T cells compared with sham exosomes and suggested that this may contribute to tumor escape mechanisms. Zhang et al. (42) showed that the exosomes from synovial fibroblasts from individuals with rheumatoid arthritis but not those from individuals with osteoarthritis, contain a membrane bound form of TNFα. These exosomes are taken up by activated T cells and induce apoptosis resistance of these cells. TNFα and IL-1β are also increased in exosomes from patients with Parkinson’s disease, which transmit pathological effects and induce motor impairment when injected to normal mice (27). TNFα in the EVs may also induce beneficial effects in certain pathologies. For example, Munich et al. (43) showed that dendritic cell exosomes contain TNFα and TNF superfamily ligands which directly kill tumor cells and activate natural killer cells. TNFα is also present in EVs released from microglia and it has been proposed that it plays a role in regulating CNS functions, including the neuroinflammatory response following brain injury, the neuronal circuit formation and synaptic plasticity (44). These reports suggest that TNFα levels are increased in EVs in different pathological situations, contributing to their propagation. We have identified here some key mechanisms by which enhanced TNFα levels in EVs in hyperammonemia induce neuroinflammation and enhance GABAergic neurotransmission in cerebellum, which would lead to motor incoordination.

In summary, this report shows that ex vivo treatment of cerebellar slices from control rats with HA-EVs induce glial activation, neuroinflammation and enhance GABAergic neurotransmission in cerebellum of control rats, reproducing the effects induced by hyperammonemia *in vivo*. Moreover, we identify in detail key mechanisms involved in the above effects. HA-EVs induce the activation of both the TNFR1-CCL2-BDNF-TrkB-KCC2 pathway and the TNFR1-NF-kB-glutaminase-GAT3 pathway. Activation of these pathways enhances GABAergic neurotransmission in cerebellum, which is responsible for the induction of motor incoordination by HA-EVs. The data also show that the increased levels of TNFα in HA-EVs are responsible for the above effects and that the activation of both pathways is prevented by blocking the action of TNFα. This opens new therapeutic options to improve motor coordination in hyperammonemia and also in cirrhotic patients with hepatic encephalopathy and likely in other pathologies in which altered cargo of EVs contribute to the propagation of the pathology.

## Data Availability Statement

The raw data supporting the conclusions of this article will be made available by the authors, without undue reservation.

## Ethics Statement

The animal study was reviewed and approved by Comite de Experimentación y Bienestar Animal (CEBA) of Principe Felipe Research Centre and by Conselleria de Agricultura of Generalitat Valenciana.

## Author Contributions

PI-A: experimental work (ex vivo experiments, immunohistochemistry and immunofluorescence staining), data analysis, and draft of the manuscript; MM-G: experimental work (extracellular vesicle isolation, Western blot); VF: obtained funding, study concept, design and supervision, interpretation of data, and writing of the manuscript. All authors contributed to the article and approved the submitted version.

## Funding

This work was supported in part by the Ministerio de Ciencia e Innovación Spain (PID2020-113388RB-I00), Consellería Educación Generalitat Valenciana (PROMETEOII/2018/051), and co-funded with European Regional Development Funds (ERDF). PI-A has a contract from Ministerio de Ciencia, Innovación y Universidades of Spain (FPU17/01698).

## Conflict of Interest

The authors declare that the research was conducted in the absence of any commercial or financial relationships that could be construed as a potential conflict of interest.

## Publisher’s Note

All claims expressed in this article are solely those of the authors and do not necessarily represent those of their affiliated organizations, or those of the publisher, the editors and the reviewers. Any product that may be evaluated in this article, or claim that may be made by its manufacturer, is not guaranteed or endorsed by the publisher.
